# Malignancy-Associated Renal Infarction: A Case of Prostate Cancer Presenting With Flank Pain and Hematuria

**DOI:** 10.7759/cureus.91430

**Published:** 2025-09-01

**Authors:** Connor J Stinson, Alec Pupo, Patrick Laird

**Affiliations:** 1 Internal Medicine, Philadelphia College of Osteopathic Medicine, Philadelphia, USA; 2 Orthopaedics, Broward Health, Fort Lauderdale, USA

**Keywords:** acute arterial thrombosis, androgen depriving therapy, flank pain, malignancy-associated hypercoagulability, prostate cancer, renal infarct

## Abstract

Renal infarction is an uncommon but critical diagnosis that is frequently overlooked due to its nonspecific presentation and overlap with more common causes of abdominal or flank pain. Patients with malignancy, including metastatic prostate cancer, are at increased risk of thromboembolic events due to the intrinsic hypercoagulable state promoted by cancer, yet arterial events, such as renal infarction, remain underrecognized.

We present the case of a 74-year-old male with metastatic castrate-sensitive prostate cancer who developed acute abdominal pain, nausea, and vomiting. Evaluation revealed elevated blood pressure, microscopic hematuria, and increased lactate dehydrogenase (LDH). CT scan of the abdomen and pelvis with contrast demonstrated wedge-shaped perfusion defects in the left kidney, consistent with renal infarction, allowing for the timely initiation of anticoagulation. This case underscores the importance of recognizing renal infarction as a potential complication of malignancy-associated thrombophilia. The classic triad of flank pain, hematuria, and elevated LDH should raise clinical suspicion, prompting early cross-sectional imaging to facilitate diagnosis and prevent irreversible renal injury.

## Introduction

Renal infarction is a rare but critical diagnosis that is often missed due to its silent presentation, nonspecific symptoms, and overlap with more common abdominal pathologies. Flank pain, hematuria, nausea, and vomiting frequently lead clinicians down diagnostic pathways for nephrolithiasis, pyelonephritis, or gastrointestinal causes, while renal infarct is a differential diagnosis that is less often considered. The true incidence of renal infarction remains underestimated, in part because non-contrast imaging, the typical first step in stone evaluation, often fails to identify perfusion defects [1].

Cancer patients live in a prothrombotic state, and while malignancy-associated venous thrombosis is well-established, arterial thrombotic events like renal infarction are less frequently recognized. Prostate cancer, particularly in advanced stages, further elevates this risk through hypercoagulability, proinflammatory cytokine release, and androgen deprivation therapy (ADT)-related metabolic changes [2,3]. Although venous thromboembolism is more commonly reported in prostate cancer, arterial events should not be overlooked, especially in patients with metastatic disease.

We report a case of renal infarction in a patient with metastatic castrate-sensitive prostate cancer, illustrating the diagnostic pitfalls and emphasizing the need for heightened clinical suspicion when encountering the classic triad of flank pain, hematuria, and elevated lactate dehydrogenase (LDH) in oncologic patients.

## Case presentation

A 74-year-old male with a history of metastatic castrate-sensitive prostate cancer, chronic mild neutropenia, and hemorrhoids presented from home with nausea, vomiting, and abdominal pain. He states his symptoms began the previous evening, around 7 pm, when he developed sharp periumbilical abdominal pain. Hours later, nausea with vomiting ensued. He reported a suspected fever at home the same day, but did not check his temperature. His symptoms persisted through the night, prompting him to seek care.

He was first diagnosed with prostate adenocarcinoma 6 years ago following a prostate biopsy with 13 of 15 positive cores. His highest Gleason Score was 8. At the time, an MRI of his abdomen revealed right pelvic lymph node involvement. He was started on leuprolide, abiraterone, and prednisone for two years of treatment. After two years, his PSA was < 0.05, and repeat imaging showed no evidence of disease progression, so he was monitored off anti-neoplastics. After three years, his prostate cancer showed evidence of local recurrence, now with metastatic retroperitoneal lymph node involvement. He was started back on leuprolide, abiraterone, and prednisone, which he had been on for approximately one year prior to presentation. These are his only current medications; he does not take anti-coagulation.

On arrival at the ED, his temperature was 98.7. He had an elevated blood pressure of 160/85, while his vital signs were otherwise stable. On exam, he had diffuse abdominal tenderness and no flank pain bilaterally. There was no edema of the lower extremities. The cardiovascular exam showed a regular rate and rhythm. ECG showed normal sinus rhythm with non-specific ST and T wave abnormalities in the precordial leads. Noteworthy labs are outlined in Table 1.

**Table 1:** Laboratory values *Creatinine reference ranges vary slightly by lab and sex; 0.6–1.3 mg/dL is typical for adult males. LDH: lactate dehydrogenase; eGFR: estimated glomerular filtration rate; AST: aspartate aminotransferase; ALT: alanine aminotransferase; UA: urinalysis

Test	Result	Reference Range	Units
LDH	218	140–280	U/L
LDH (15 hr later)	593	140-280	U/L
Creatinine	1.18	0.6–1.3 (M)*	mg/dL
eGFR	64	>90	mL/min/1.73 m^2
AST	21	15-37	U/L
ALT	21	12-78	U/L
Alk Phos	94	46-116	U/L
Total Br	0.5	0.2-1	mg/dL
Troponin	6	< 22	ng/L
Lipase	12	0–160	U/L
UA – RBCs	0–2	0–3	/HPF
UA – Blood	Trace	Negative	—
UA – Bacteria	Occasional	None	—
UA – WBCs	0–5	0–5	/HPF

**Figure 1:**
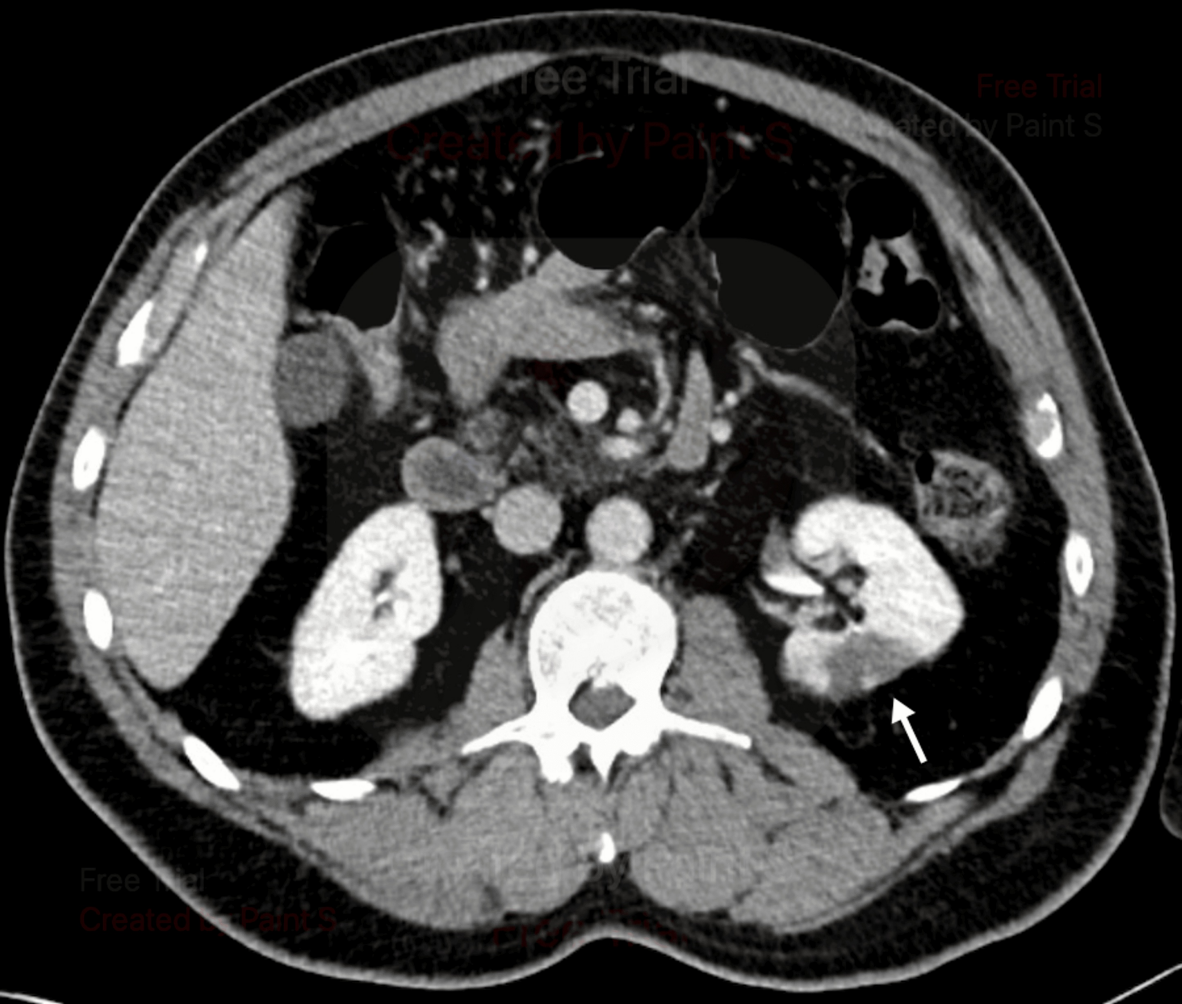
Left kidney renal infarct (white arrow) Wedge-shaped renal infarct of the left kidney

**Figure 2:**
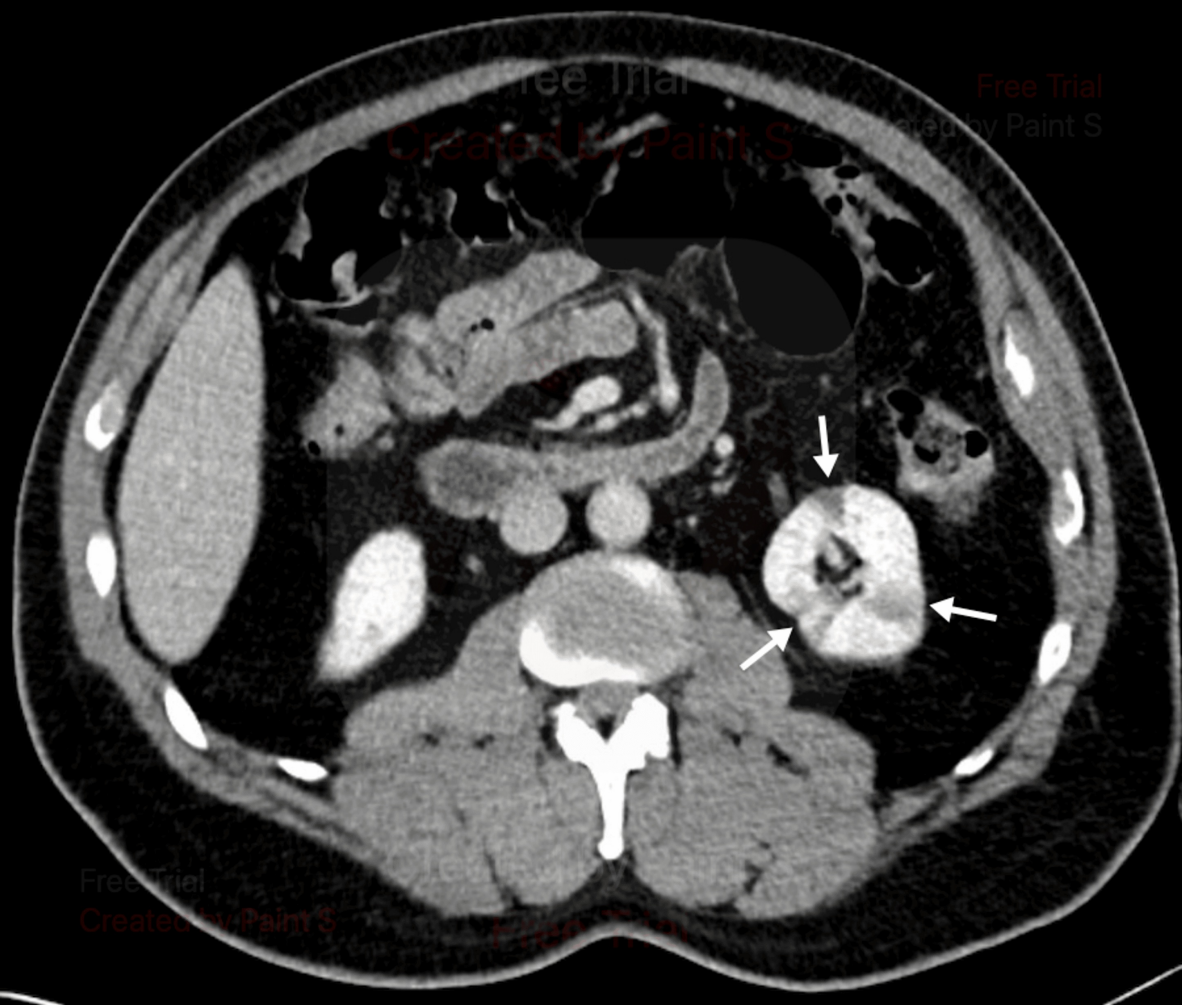
Multiple renal infarcts of the left kidney (white arrows)

Pyelonephritis was considered but thought less likely due to the patient’s lack of documented fever, absent leukocytosis, and non-infectious appearing urinalysis. Nephrolithiasis was considered, given the patient’s sharp abdominal pain, nausea with vomiting, and faint blood in the urine. Our leading differential diagnosis was renal infarction, given the nature of the patient’s symptoms, elevated blood pressure, elevated LDH, and microscopic hematuria. Ultimately, a CT abdomen pelvis with contrast was completed, showing wedge-shaped areas of low perfusion in the left kidney (Figures 1, 2).

The patient was determined not to be a candidate for emergent revascularization due to low perceived benefit. He was started on a heparin drip. Prior to discharge, he was transitioned to Eliquis 10 mg twice daily for seven days, followed by Eliquis 5 mg twice daily for three months, followed by prophylactic anticoagulation with Eliquis 2.5 mg twice daily indefinitely. 

## Discussion

Renal infarction arises when arterial blood flow to a segment of the kidney is acutely disrupted, due to an embolic or thrombotic event, leading to ischemia and tissue necrosis [4]. The incidence of renal infarction is rare, with old ER studies showing rates of 0.0004% to 0.0007%. Though autopsy studies suggest an incidence of closer to 1.4% (14 per 1000), indicating many cases are diagnosed late or misdiagnosed [4]. Patients with underlying malignancy have an intrinsically hypercoagulable state that serves as a major risk factor. In this case, we can presume our patient’s prostate cancer precipitated a hypercoagulable state through several established mechanisms. Many prostate cancers constitutively express tissue factor, which activates the extrinsic coagulation cascade, resulting in increased thrombin and fibrin production. Prostate cancer also produces a protease enzyme called cancer procoagulant, which directly activates factor X, further amplifying the combined coagulation cascade [2]. Cancer cells also secrete proinflammatory cytokines, such as interleukin-1 beta (IL-1β), tumor necrosis factor-alpha (TNF-α), and vascular endothelial growth factor (VEGF), which foment a pro-thrombotic environment in the blood [2]. It should even be noted that, though rare, tumor emboli (small fragments of tumor cells or thrombus from tumor-laden vessels) can break off and lodge themselves in small vessels such as the renal arteries and arterioles. Case reports have described this phenomenon causing renal infarct with lung cancer [5], though my search yielded no proven occurrences resulting from prostate cancer.

Taking a look beyond the pathophysiology, these mechanisms have been demonstrated by clinical studies quantifying the increased risk of thromboembolic events in patients with cancer. Prostate cancer has a relatively lower associated risk of venous thromboembolism (VTE) compared to other cancers like pancreatic, colorectal, lung, and brain [6]. However, the risk of VTE increases 1.4 to 21.5-fold higher with more advanced cancer stages, such as in our patient with metastatic disease [3]. Castrate-sensitive refers to prostate cancer that remains responsive to androgen-deprivation therapy (ADT); this distinction is critical, as castrate-sensitive disease is treated with androgen deprivation therapy (ADT), whereas castrate-resistant disease requires alternative systemic therapies. Observational studies of ADT (most commonly utilized: leuprolide) have also been associated with increased arterial and venous thromboembolism risk, up to 84% increased risk in some studies [7]. The mechanism is believed to be due to ADT’s testosterone-lowering effect, as low testosterone in men has been associated with reduced fibrinolytic activity in clinical studies [7]. 

Clinically, renal infarction commonly presents with abrupt flank or abdominal pain, often accompanied by microscopic or gross hematuria, reflecting necrosis of renal parenchyma [4]. Hematuria is present in up to 32% of patients presenting with renal infarction. Hypertension is another common finding at presentation. Reduced perfusion within the ischemic renal segment prompts renin release from juxtaglomerular cells, activating the renin-angiotensin-aldosterone system (RAAS) and elevating systemic blood pressure. Approximately 48% of patients exhibit elevated blood pressure at presentation, and malignant hypertension has been documented in up to 22% [4].

Laboratory values typically show a striking elevation in serum LDH, as massive cellular destruction releases intracellular enzymes. Often, LDH levels can exceed four times the upper limit of normal, helping distinguish infarction from more benign causes of flank pain such as nephrolithiasis or pyelonephritis [4]. Elevated LDH without a significant transaminase rise can help distinguish renal infarction from hepatic ischemia [4]. On admission lab tests, our patient's LDH was only mildly elevated at 218. This could indicate less extensive ischemia or a partial infarct. In this case, however, it likely indicates detection early in the disease course, as repeat LDH 15 hours later had increased to 593. Our patient’s elevated LDH was the pathognomonic clue leading us away from our alternative differentials and in favor of renal infarct.

After the diagnosis of renal infarct was secured in our patient, Interventional Radiology was contacted to evaluate for the potential benefit of revascularization therapy. Because of his only mildly diminished renal function and patency of the renal artery on imaging, it was determined that there was likely little benefit to revascularization techniques. Some methods of revascularization that are utilized as standard practice include: endovascular thrombectomy, local thrombolysis, angioplasty, and stent placement. Systemic fibrinolytic therapy may be used in settings where endovascular intervention is not available (provided dissection is not the cause of infarct); however, there is limited data to support this approach [8]. The window for the revascularization of renal infarction is not strictly defined in the literature. However, as a maxim, earlier intervention is preferred. In one study utilizing catheter-directed local thrombolytic therapy, revascularization was performed after presentation, for up to 24 hours or more [9]. Systemic anticoagulation is considered a keystone element of treatment for renal infarction. Choice and duration of anticoagulant (e.g., apixiban, warfarin) is directed by the underlying condition. Workup for thromboembolic risk factors (atrial fibrillation, vascular abnormality, hypercoagulable disorder, etc.) should be completed in all new cases. Patients with an identified risk factor are often treated with anticoagulation alone (sometimes indefinitely). Patients who undergo angioplasty, stent placement, or those who have an underlying vascular abnormality are typically also treated with an antiplatelet agent such as aspirin or clopidogrel [8].

Renal infarction is often misdiagnosed or overlooked due to its nonspecific presentation and clinical overlap with more common conditions such as nephrolithiasis, pyelonephritis, diverticulitis, or musculoskeletal pain. Symptoms like acute flank pain, hematuria, and leukocytosis are frequently attributed to these alternatives, particularly when non-contrast CT, commonly used for stone evaluation, misses perfusion defects. Key diagnostic clues, including a markedly elevated LDH and sudden-onset hypertension, are often underrecognized [1]. In fact, many patients with renal infarction are asymptomatic, and diagnosis is made solely by diagnostic imaging. Early diagnosis requires a high index of suspicion in patients with thromboembolic risk factors (in this case, malignancy). When renal infarction is suspected, CT angiography is the imaging modality of choice, allowing direct visualization of vascular perfusion of renal parenchyma. Prompt recognition is critical, as delayed treatment may lead to irreversible renal injury and missed opportunities for anticoagulation or kidney-preserving vascular intervention.

## Conclusions

The classic triad of flank pain, hematuria, and elevated lactate dehydrogenase (LDH) should raise clinical suspicion for renal infarction. This case report aims to increase awareness of renal infarction as a diagnostic consideration in patients with underlying malignancy. The prothrombotic state associated with cancer significantly elevates the risk of arterial thrombosis, including infarction of the renal vasculature. Given that many patients, such as in this case, may present with only one or two of the hallmark findings or remain entirely asymptomatic, clinicians must maintain a high index of suspicion and a low threshold to obtain early cross-sectional imaging (CT-angiography), particularly in oncologic patients, where the risk for ischemic vascular events is inherently higher.

## References

[1] Scarpioni R, Michieletti E, Cristinelli L (2005). Atherosclerotic renovascular disease: medical therapy versus medical therapy plus renal artery stenting in preventing renal failure progression: the rationale and study design of a prospective, multicenter and randomized trial (NITER). J Nephrol.

[2] Chew HK, Wun T, Harvey D, Zhou H, White RH (2006). Incidence of venous thromboembolism and its effect on survival among patients with common cancers. Arch Intern Med.

[3] Walker AJ, Card TR, West J, Crooks C, Grainge MJ (2013). Incidence of venous thromboembolism in patients with cancer - a cohort study using linked United Kingdom databases. Eur J Cancer.

[4] Mulayamkuzhiyil Saju J, Leslie SW (2024). Renal infarction. StatPearls [Internet].

[5] Hussain SM (2023). Lung cancer embolization causing acute limb ischemia: a case report. J Med Case Rep.

[6] Caine GJ, Stonelake PS, Lip GY, Kehoe ST (2002). The hypercoagulable state of malignancy: pathogenesis and current debate. Neoplasia.

[7] Klil-Drori AJ, Yin H, Tagalakis V, Aprikian A, Azoulay L (2016). Androgen deprivation therapy for prostate cancer and the risk of venous thromboembolism. Eur Urol.

[8] Bhalla V, Textor SC, Beckman JA (2022). Revascularization for renovascular disease: a scientific statement from the American Heart Association. Hypertension.

[9] Silverberg D, Menes T, Rimon U, Salomon O, Halak M (2016). Acute renal artery occlusion: presentation, treatment, and outcome. J Vasc Surg.

